# Multifunctional Proteins: Involvement in Human Diseases and Targets of Current Drugs

**DOI:** 10.1007/s10930-018-9790-x

**Published:** 2018-08-19

**Authors:** Luis Franco-Serrano, Mario Huerta, Sergio Hernández, Juan Cedano, JosepAntoni Perez-Pons, Jaume Piñol, Angel Mozo-Villarias, Isaac Amela, Enrique Querol

**Affiliations:** 1 Departament de Bioquímica i Biologia Molecular and Institut de Biotecnologia i Biomedicina, Universitat Autònoma de Barcelona, 08193 Cerdanyola del Vallès, Barcelona, Spain; 2 Laboratorio de Inmunología, Universidad de la República Regional Norte-Salto, Rivera 1350, 50000 Salto, Uruguay; 3 Departament de Medicina Experimental and Institut de Recerca Biomèdica, Universitat de Lleida, 25198 Lleida, Spain

**Keywords:** Multitasking proteins, Human diseases, Protein function, Drug targets

## Abstract

Multifunctionality or multitasking is the capability of some proteins to execute two or more biochemical functions. The objective of this work is to explore the relationship between multifunctional proteins, human diseases and drug targeting. The analysis of the proportion of multitasking proteins from the MultitaskProtDB-II database shows that 78% of the proteins analyzed are involved in human diseases. This percentage is much higher than the 17.9% found in human proteins in general. A similar analysis using drug target databases shows that 48% of these analyzed human multitasking proteins are targets of current drugs, while only 9.8% of the human proteins present in UniProt are specified as drug targets. In almost 50% of these proteins, both the canonical and moonlighting functions are related to the molecular basis of the disease. A procedure to identify multifunctional proteins from disease databases and a method to structurally map the canonical and moonlighting functions of the protein have also been proposed here. Both of the previous percentages suggest that multitasking is not a rare phenomenon in proteins causing human diseases, and that their detailed study might explain some collateral drug effects.

**Electronic supplementary material:**

The online version of this article (10.1007/s10930-018-9790-x) contains supplementary material, which is available to authorized users.

## Introduction

The aim of this work was to analyse the link between moonlighting proteins and human diseases, as well as between moonlighting proteins and current drug targets. Moonlighting or multitasking proteins are those proteins with two or more biochemical functions performed by a single polypeptide chain. Wistow and Piatigorsky discovered them three decades ago when they demonstrated that *lens crystallins* and some metabolic enzymes were the same protein, even doing a completely different function and in distinct cellular localizations [1]. Piatigorsky proposed the term *gene sharing* for these proteins [2]. The term *moonlighting* was used for the first time by Constance Jeffery [3] who intended to reach a more restrictive definition, as this term does not include the cases of gene fusions. Moonlighting proteins present alternative functions, usually related to cellular localization, cell type, oligomeric state, concentration of cellular ligands, substrates, cofactors, products or post-translational modifications [2–16]. In many cases, a protein uses a combination of these mechanisms to switch between functions. In the present work, the different functions of multitasking proteins have been labelled as “canonical” or “moonlighting”, but this has no biological relevance and merely refers to the chronological order of the discovery of the biological function, the first being canonical [17]. The fact that it is an “archaic” function or a function of “recent” acquisition affects some aspects of the function, in relation to factors such as the contribution to the pathology. Even the fact that some prediction methods, like for example domain or motif searches, can easily detect the canonical function, but they have serious problems in finding the moonlighting function. For this reason, seems interesting to continue with this terminology that, moreover, other authors in the field maintain. Nevertheless, there are authors that use *Function 1* and *Function 2* instead of canonical and moonlighting [18]. In general, moonlighting proteins are experimentally revealed by serendipity. We only know a small part of the existing moonlighting proteins. As initially written by Jeffery [6], this type of proteins “appear to be only the tip of the iceberg”.

Several authors have reported a number of moonlighting proteins involved in human disease [7–9], but, as far as we know, there is not a database combining moonlighting proteins and human diseases. In the present work, it is reported the percentage of human moonlighting proteins that are involved in diseases or are targets of current drugs. Also, a procedure is described to identify putative moonlighting proteins from disease databases. When the three-dimensional structure of the protein is available, a method to structurally map the canonical and moonlighting functions of the protein is also proposed. In some cases, these studies can be combined with the aim of explaining some collateral effects of a drug related to this moonlighting protein. It has to be taken into account that, although bioinformatics analyses can help to suggest which proteins are multifunctional and, in some cases, map the two functional sites in the structure of the protein, identifying true positives must always be demonstrated experimentally.

## Materials and Methods

### Databases

Moonlighting proteins listed in the three currently existing databases that contain experimentally determined multitasking proteins have been used. These databases are: MultitaskProtDB-II [17], MoonProt 2.0 [18] and MoonDB [19] and are accessible at http://wallace.uab.es/multitask, http://www.moonlightingproteins.org and http://tagc.univ-mrs.fr/MoonDB, respectively. The information present in the Human Mendelian Inheritance in Man (OMIM, http://www.omim.org) [20] database and the Human Gene Mutation Database (HGMD, http://www.hgmd.cf.ac.uk) [21] for each of the proteins has been carefully inspected. With this strategy, the moonlighting proteins that are involved in human diseases were identified. Moreover, the Therapeutic Target Database (TTD, ttp://bidd.nus.edu.sg/group/cjttd) [22] and the DrugBank Database (http://www.drugbank.ca) [23] have been scanned for relevant information in order to see if each of the moonlighting proteins is a drug target. When necessary, some important protein characteristics have been retrieved from The UniProt Consortium (http://www.UniProt.org) [24]. If available, the three-dimensional structure of the protein has been obtained from The Protein Data Bank (http://www.rcsb.org) [25].

Statistical significance of the data obtained from these sources was analysed by the ODD ratio. The confidence interval (CI) used was of 95%. The ODD ratio is a standard system to measure the degree of association between categorical variables of two states (in our case disease/no-disease vs. moonlighting/no-moonlighting and druggable/no-druggable vs. moonlighting/no-moonlighting). In order to establish the statistical significance of the observed differences the Fisher’s exact test has been calculated using R.

### Prediction of New Moonlighting Protein Candidates Using OMIM

**Fig. 1:**
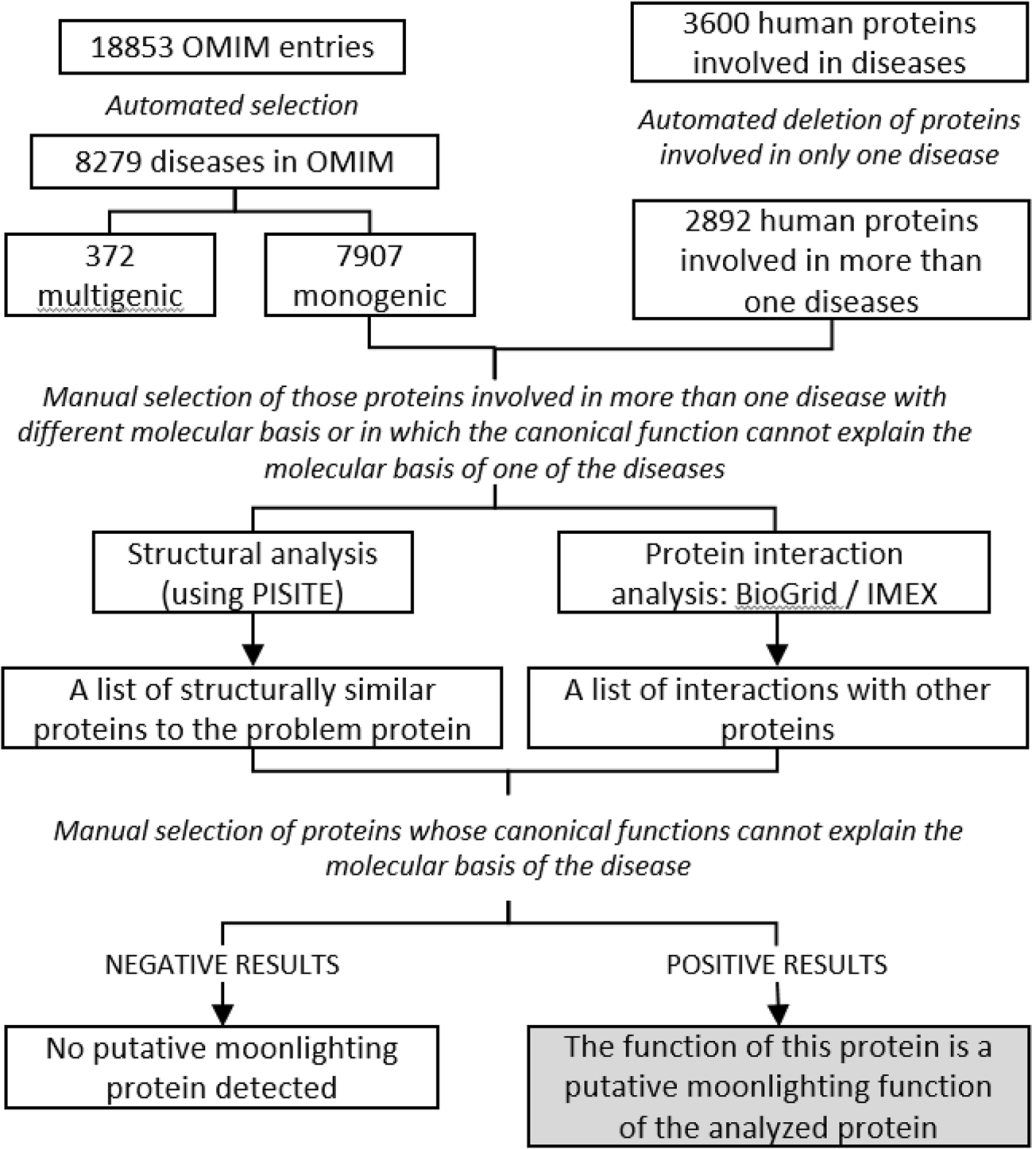
Chart flow representation of the process followed to predict moonlighting proteins using OMIM database, structural analysis and protein interaction

With the objective of crossing the information between the UniProt and the OMIM databases, this sequential procedure has been followed: (a) make a list of UniProt proteins that are related to diseases according to the OMIM database and the literature (3600 proteins were initially collected); (b) remove the disease entries caused by more than one protein; (c) select only those proteins that cause more than one disease; (d) manually review the diseases that are caused by each of these proteins and: (d1) select those proteins in which the diseases are not related to each other, or (d2) select those proteins in which the diseases do not seem to be related (this means a different molecular basis) to the canonical function of the putative moonlighting protein. This final list should contain entries which can be characterized as putative moonlighting proteins (Fig. 1).

**Fig. 2:**
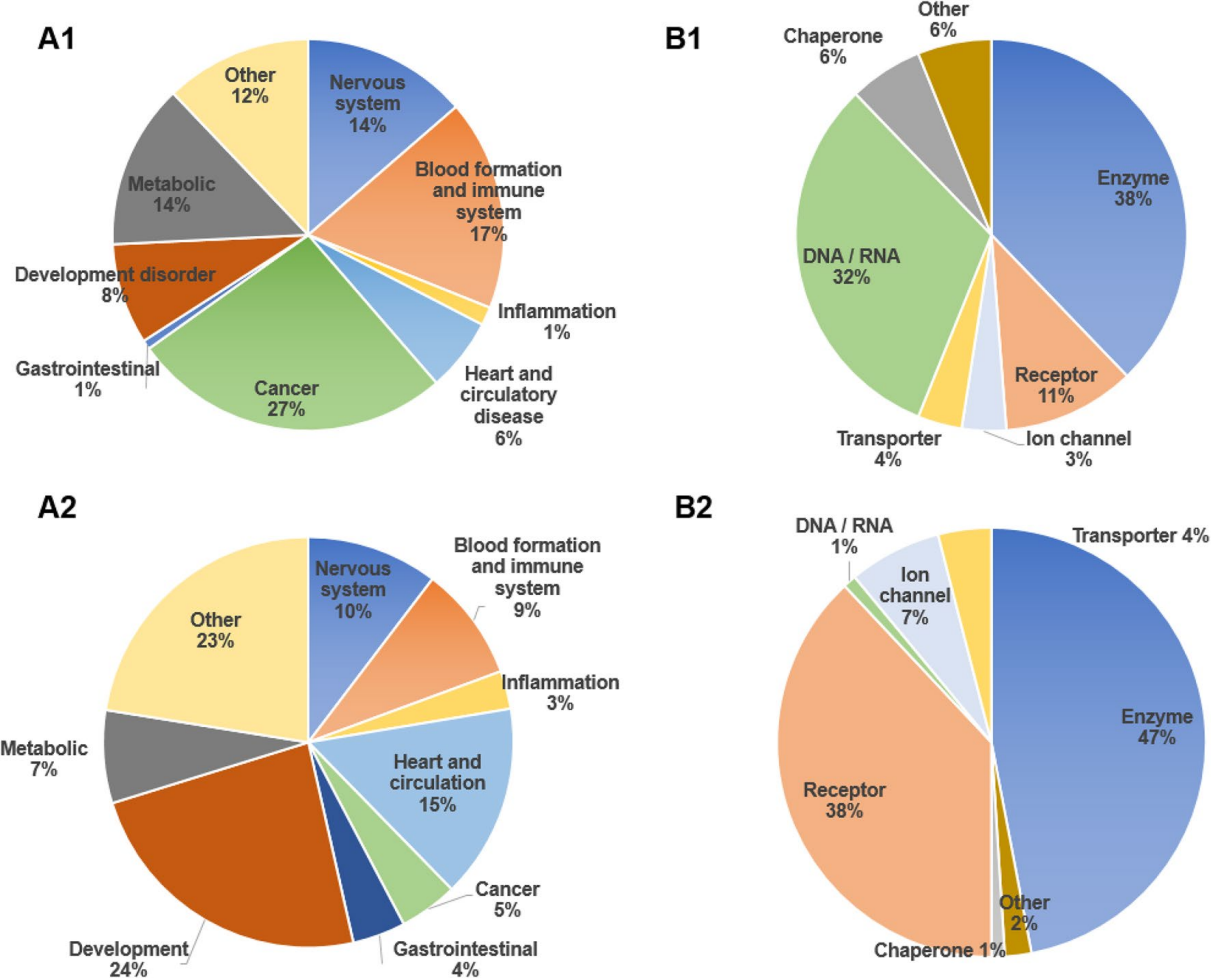
**A1** Distribution of disorders associated with human moonlighting proteins and their relative percentages. **A2** Distribution of disorders associated to human proteins, in general. **B1** Functional classification of drug-target moonlighting proteins and their relative percentages. **B2** Functional classification of drug-target in general, and their relative percentages

### Mapping and Linking Diseases to the Canonical or Moonlighting Function

An exhaustive analysis of the literature related to the diseases associated with the human multitasking proteins from the MultitaskProtDB-II (S1 Table) was performed. For each of these cases, the mechanism of action of the pathology has been studied with the aim of relating at a molecular level, the disease to the canonical, moonlighting or both functions. In some of them, not enough data could be found in the literature in order to relate the pathology to one of the functions. When relevant data exist and to demonstrate this condition and try to map the canonical and moonlighting functions in the structure of the protein, the use of a combination of different methods has been proposed. If the three-dimensional structure of the protein is available, the program PiSite [26] can be used, as reported by our group in other studies [27, 28]. This algorithm searches for proteins with a similar three-dimensional structure to the query protein. The results should again be manually reviewed and used to find for protein structures that can explain the disease that is not caused by the canonical function. In these cases, the function done by this new protein might be the moonlighting function of the original protein. With no three-dimensional structure of the protein, protein interaction databases, such as BioGRID [29] or IMEX consortium [30], can be used. In this case, we can look for proteins that interact with our protein and that can explain the disease not related to the canonical function of the query protein. In addition, this interacting protein might have an already-solved three-dimensional structure and, therefore, the PiSite method mentioned before can be applied again. Beginning with the structure of the putative moonlighting protein or with the structure of a closely related interacting protein, the structural annotations can be obtained from the literature and UniProt. This information contains the key functional regions and amino acids of the protein, which should be used to link these characteristics to the pathological effect of the disease. Finally, these important regions should be localized in the structure of the protein, verifying that they are in different zones in order to map the canonical and moonlighting functions. A schematic flow-chart has been created to clearly explain the process (Fig. 1). It has to be taken into consideration that in all steps of the procedure, a more or less detailed manual inspection is required in order to obtain significant results.

Our group has collected by the date 611 multitasking proteins. This number of proteins doubles the entries of MultitaskProtDB [17] and can be found in S1 Table. The results presented here have been calculated in reference to this new number.

## Results and Discussion

### Moonlighting Proteins and Human Diseases

The first analysis performed was to find how many human moonlighting proteins are related to known human diseases. An exhaustive analysis of the literature related to the diseases associated with the human multitasking proteins of the MultitaskProtDB-II (S1 Table) shows that 78% of the moonlighting proteins in MultitaskProtDB-II are related to diseases. These proteins with a description of their functions and the diseases in which they are involved can be seen in S2 Table.

The number of human proteins indicated as reviewed entries in the UniProt database is 20,168 at the time of the study. On the one hand, from the OMIM database we saw that only 3600 of these proteins are related to human diseases. This result is clearly significant, as can be seen in the S4 Table, and it represents a percentage of 17.85%. On the other hand, a set of the currently determined human moonlighting proteins was created using the published MultitaskProtDBII, which can be found in the S1 Table. As before, we checked in OMIM and in the literature if these proteins were related with human diseases, and a surprising number was found that shows that 78% of the analyzed proteins are involved in human diseases. This percentage is much higher than the 17.85% found in human proteins in general. The probabilities that a human-UniProt-protein and a human -MultitaskProtDB-protein were involved in a known OMIM-disease, were calculated using the ODD ratio. Altogether, these results suggest that moonlighting proteins are prone to be involved in human diseases as it is pointed by it respective ODD ratios; 16.47 (CI 95% 10.95–25.44) in the OMIM analysis being highly significant (Fisher’s exact test value; p < 2.2e-16) (See S6 Table).

Some relevant examples of moonlighting proteins that are involved in human diseases can be seen in Table 1. They were identified after crossing the data of OMIM and those of HGMD. These examples have been chosen to show cases in which the phenotype can be easily attributable to one of the biological functions of the protein. In bold, the putative functions involved in diseases are indicated: (C) for those diseases related to the canonical function and (M) for those related to the moonlighting function. There are some examples where each function is related to a different disease (i.e., Fumarate hydratase). Sometimes it is difficult to elucidate which function is responsible for the disease, suggesting that both functions might contribute to the different symptoms (i.e., Hes1 protein). A huge list of the entire set of 112 moonlighting proteins that are involved in human diseases can be found in the S2 Table. A pie-chart representation showing the percentages of moonlighting proteins classified by type of disease can be seen in Fig. 2A1. These results could be compared with Fig. 2A2 that shows the same data but related to the entire set of human proteins involved in diseases according to Uniprot [24]. Two recent works belonging to Brun’s group predict 3% of the human interactome correspond to moonlighting proteins. They also say that these proteins are significantly involved in more than one disease or comorbidity [19, 31]. Fourteen of their set of predicted human moonlighting proteins can be found in our list of moonlighting proteins involved in human diseases (S2 Table). It has to be considered that our list was only made up of experimentally demonstrated multitasking proteins, while a number of the moonlighting proteins of Brun’s group’s studies correspond to predicted moonlighting proteins [32]. The above results imply that human moonlighting proteins are significantly associated to human diseases compared to non-moonlighting ones.

**Table 1:** Examples of moonlighting proteins involved in human diseases and drug targets

Protein name (*)	Canonical function	Moonlighting function	Disease	Molecular process reference (*)	Drug targets (*)
Cyclooxygenase 1	Prostaglandin G/H synthase	Heme-dependent peroxidase	(C) Bleeding disorder type 12	Brit. J. Haemat. 92: 212–217, 1996	YES
Gephyrin, protein anchor	Microtubule-associated protein	Synthesis of molybdenum cofactor (MoCo**)**	(M) Molybdenum cofactor deficiency C	Am J Hum Genet. 2001 Jan;68(1):208–13	YES
Ribosomal S3 protein	Ribosomal protein	DNA repair	(M) Colon adenocarcinomas	10.1016/j.tig.2014.06.003	NO
Succinyl-coA synthetase	Succinyl-CoA synthetase	Mitochondrial DNA maintenance	(M) Mitochondrial DNA depletion syndrome 9 (encephalomyopathic type)	J. Med. Genet. 47: 670–676, 2010	YES
Fumarate hydratase	TCA cycle	Tumor suppressor	(C) Fumarase deficiency (M) Leiomyomatosis with Renal cell cancer	Oncogene. 2015 Mar 19;34(12):1475–86	NO
ERCC2—TFIIH	DNA helicase DNA repair damaged by exposure to ultraviolet light	It is also a subunit of TFIIH, a basal transcription factor	(C) Xeroderma pigmentosum (C) Cerebrooculo facioskeletal syndrome 2	- Mutat Res. 1992 Mar;273(2):193–202 - Am. J. Hum. Genet. 69: 291–300, 2001	NO
Alpha-crystallin A chain	Lens crystallin	Heat-shock protein	(C) Cataract (M) Autoimmune diseases (C) Uveitis	Biomed Pap Med Fac Univ Palacky Olomouc Czech Repub. 2005 Dec;149(2):243–9	YES
Hes 1 protein	Transcriptional repressor	It is able to induce the activation of the NF-kB pathway in T Cell Leukemia	(M) Leukemia, myeloid/lymphoid or mixed-lineage	Cancer Cell. 2004 Sep;6(3):203–8	NO
PIAS1	Inhibition of activated STAT	Activation of p53	(M) Cancer	Cold Spring Harb Perspect Biol. 2009 Nov; 1(5): a001883	NO
Phosphoglucose isomerase	Glycolysis	Neuroleukin, differentiation and maturation factor/nerve growth factor/stimulation of cell migration/implantation factor/modulator of tumor progression and a target for cancer therapy/sperm surface antigen	(C) Hemolytic anemia PGI deficiency (C) Angiogenesis in cancer	- Harefuah. 1994 Jun 15;126(12):699–702, 764, 763 - Cancer Res 2003;63:242–249	YES

*C* Disease related to canonical function, *M* Disease related to moonlighting function

*Entries are linked to the corresponding information

### Prediction of Putative New Moonlighting Protein Candidates Using OMIM

In general, moonlighting proteins are experimentally revealed by serendipity. Thus, as far as possible, it would be very interesting identifying them bioinformatically. Several attempts to bioinformatically predict multitasking proteins have been proposed by the teams of Brun [19, 32], Kihara [33–35] and ours [27, 28, 35–37]. One interesting question that occurred to us is whether human genetic-disease databases, such as OMIM, could be a useful resource to find moonlighting proteins. A manual inspection of human disease databases was started to disclose some putative moonlighting proteins and, moreover, to try to suggest the molecular basis of the associated disease. Table 2 shows some examples of the prediction of putative moonlighting proteins present in the OMIM database. It has to be considered that the data of protein–protein interaction (PPI) databases and the use of the structure comparison tool PiSite can help in explaining the unexpected relation with the canonical/moonlighting function and the associated disease. For example, in the case of the Fanconi anemia group J protein (Q9BX63), PPI database searches show that this protein interacts with DNA repair proteins but also with the breast cancer protein BRC1. Another example is the calcium-independent phospholipase A2 (O60733), in which the canonical function of the protein is related to fatty acid metabolism, but it seems to be also involved in brain neurodegenerative diseases. Moreover, a PPI analysis shows a relation between this protein and BAG, a protein involved in apoptosis and cell survival. What is more, using PiSite, we found whose structure is similar to the apoptosis protein caspase-2. More examples on how moonlighting proteins can be predicted using disease databases, together with structural and interactomics analyses are shown in Table 2.

**Table 2:** Examples of predicted human moonlighting proteins and their associated genetic diseases

Protein and UniProt descriptors (*)	Canonical (C) and predicted moonlighting functions (M)	Associated diseases and reference (*)	Interactomics partners (*)	PISITE models (*)
3-hydroxyacyl-CoA dehydrogenase type-2 Q99714 HCD2_HUMAN	(C) mitochondrial ribonuclease P (M) beta-oxidation of fatty acids	(1) 2-methyl-3-hydroxybutyryl-CoA dehydrogenase deficiency (MHBD deficiency) (2) Mental retardation X-linked, syndromes	(1)Amyloid beta A4 protein (2) Mitochondrial ribonuclease P protein 1 (3) Sulfatase-modifying factor 1 (4) Mitochondrial ribonuclease P protein 3	(1) 3-hydroxyacyl-CoA dehydrogenase type-2 PDBID: 1so8 CHAIN:A (2) 3-alpha-(or 20-beta)-hydroxysteroid dehydrogenase PDBID: 1nfq CHAIN:C
Pyruvate kinase PKLR P30613 KPYR_HUMAN	(C) Glycolysis (M) May participate in red cell survival	(1) Pyruvate kinase hyperactivity (PKHYP) (2) Pyruvate kinase deficiency of red cells (PKRD)	(1) Myocilin (2) Kinesin-like protein KIF23 (3) Rho guanine nucleotide exchange factor 7 (4) Rho guanine nucleotide exchange factor 6 (5) Paxillin (6) Serine/threonine-protein kinase PAK 1	No relevant matches found
Fanconi anemia group J protein Q9BX63 FANCJ_HUMAN	(C) DNA-dependent ATPase and 5′ to 3′ DNA helicase (M) Involved in the repair of DNA double-strand breaks	(1) Breast cancer (BC) (2) Fanconi anemia complementation group J (FANCJ)	(1) Breast cancer type 1 susceptibility protein (2) DNA mismatch repair protein Mlh1 (3) Mismatch repair endonuclease PMS2 (4) POZ-, AT hook-, and zinc finger-containing protein 1	No relevant matches found
85/88 kDa calcium-independent phospholipase A2 O60733 PLPL9_HUMAN	(C) Catalyzes the release of fatty acids from phospholipids (M) May participate in apoptosis	(1) Neurodegeneration with brain iron accumulation 2B (NBIA2B) (2) Neurodegeneration with brain iron accumulation 2A (NBIA2A) (3) Parkinson disease 14 (PARK14)	(1) BAG family molecular chaperone regulator 3	(1) CASPASE-2 PDBID: 2p2c CHAIN:U
Alpha-aminoadipic semialdehyde synthase, mitocondrial Q9UDR5 AASS_HUMAN	(C) Lysine-ketoglutarate reductase (M) Saccharopine dehydrogenase	(1) Hyperlysinemia, 1 (HYPLYS1) (2) 2,4-dienoyl-CoA reductase deficiency (DECRD)	(1) mRNA-decapping enzyme 1A (2) Peptidyl-tRNA hydrolase ICT1, mitochondrial (3) Myc proto-oncogene protein (4) Telomeric repeat-binding factor 2	(1) SACCHAROPINE DEHYDROGENASE PDBID: 2axq CHAIN:A (2) SACCHAROPINE REDUCTASE PDBID: 1e5q CHAIN:H

*Entries are linked to the corresponding information

An intriguing question is whether the mutants of the interaction partners of a disease-related moonlighting protein can also be the cause of the same, or at least very similar, pathology. This idea strongly reinforces the involvement of these mutants in the ailment. Regarding this issue, there are some examples in Table 2, such as 3-hydroxyacyl-CoA dehydrogenase. This protein is involved in a mental retardation disorder and its interaction partner, the amyloid beta A4 protein mutant, is involved in two cerebral-related diseases such as Alzheimer and cerebral angiopathy. Additionally, as mentioned in the previous paragraph, the interaction of phospholipase A2 with BRC1 is a good example of two partner mutants causing the same disease as that of the predicted moonlighting protein. Otherwise, interaction partners involved in different diseases can be found, suggesting that the predicted moonlighting protein may participate in another as yet unassociated disease. An example of this case is the Alpha-aminoadipic semialdehyde synthase (Q9UDR5), which presents two cancer-related interaction partners: the Myc proto-oncogene protein and the Telomeric repeat-binding factor 2.

In summary, it can be said that the prediction of moonlighting proteins using OMIM and HGMD databases can improve our knowledge at a molecular level of the clinical basis of a number of diseases. Further applications of all of these studies might help to revisit and reinterpret, some human disease phenotypes, generating new therapeutic strategies. Moreover, some drug off-side effects might be explained [38].

### A Number of Moonlighting Proteins are Drug Targets

Current human clinics require identifying the molecular basis of a disease and designing the proper therapy for it. In most of the cases the therapeutic process requires the use of drugs as the main or the complementary treatment. We have checked to what extent human moonlighting proteins are known targets of current drugs (which represent a small and biased set of the potential universe of the druggable genome) [39].

Something very curious is that 48% of the human moonlighting proteins are current drug targets, while only 9.8% of the human proteins present in UniProt are specified as drug targets. These calculations were performed as explained in the paragraph “Moonlighting proteins and human diseases”, but here we took into consideration the 1969 human proteins being drug targets present in TTD and DrugBank databases [22, 23]. This result is again clearly significant as can be seen in the S5 Table. Furthermore, the probabilities that a human -UniProt- protein and a human -MultitaskProtDB- protein are drug targets listed in TTD and DrugBank databases, were calculated using the ODD Ratio, observing an increased ratio of the proportion of druggble proteins 8.49 (CI 95% 5.99–12.00) in the moonlighting subset, being this difference highly significant (Fisher’s exact test value; p < 2.2e-16) (See S6 Table).

These calculations and the percentages above highlight the interest of moonlighting proteins for gaining insight into the molecular basis of genetic-based diseases and for rational drug-design upon target identification.

In the last column of Table 1, some examples of those related current drugs can be seen following the corresponding link. In S3 Table, the entire set of 68 moonlighting proteins currently identified as drug targets, with the corresponding references to the human diseases and drug databases, are listed. Figure 2B1 shows a pie-chart representation with the percentages of the moonlighting proteins that are drug targets classified by functional classes. These results could be compared with Fig. 2B2 that shows the same data, but related to the entire set of human proteins which are current drug-targets, according to DrugBank database [23]. Considering that many diseases involve moonlighting proteins, this should also be the same for the protein drug targets. Even so, this should not be as simple as it seems. “Druggable” does not mean being a drug target because targets of current drugs represent only successful cases [40]. Moreover, it is well known that many non-druggable targets exist. It has to be considered that Drews [39] estimated the number of potential drug targets as being between 5000 and 10,000.

Those two stated categories, human disease involvement and drug targets, do not appear mutually-exclusive, but in our analysis we have used the data of TDT and DrugBank databases, which are accepted to be of true drug targets. However, there might be information of several hidden targets in these databases, both for some specific proteins or from entire biochemical pathways. It is also true that a number of drugs can target proteins that, as far as known in the present state of the art, are not directly involved in diseases, thus contributing to mechanisms such as polypharmacology, off-target effects, etc. In a certain number of cases, these proteins are not druggable because they are involved in such number of pathways that the effect of the putative drug would be even deleterious. The biochemical and pharmacokinetic/pharmacodynamics mechanisms involved in these situations, are frequently disclosed with further deeper studies. Network pharmacology is an area now growing dramatically and, in fact, the procedure described here to map the different diseases and putative targets in a protein could contribute to gaining insight into the question raised by the reviewer. For example, the disclosing of a moonlighting, function can suggest new biochemical pathways involved in the disease that were previously hidden.

This 48% of human moonlighting proteins being drug targets, together with the fact that 78% of the OMIM genes correspond to moonlighting proteins, supports the opinion shared by many authors that moonlighting is not a rare phenomenon and, therefore, many human proteins would be multitasking. In a previous paragraph, the inspection of disease gene databases in order to disclose multitasking proteins has been suggested, and we also suggest mining moonlighting proteins for drug target-screening. One interesting question is whether human genetic-disease databases, such as OMIM, could be a useful resource to find moonlighting proteins, and whether they relate with pathologies.

### Mapping the Canonical and Moonlighting Functions

Generally, the discovery of a moonlighting protein is reported without linking each function to a specific domain of the protein. Even one of the *top* moonlighting proteins, glyceraldehyde 3-phosphate dehydrogenase, has not been functionally mapped (except for its canonical function). Therefore, it would be very useful to localize each function, as far as possible, in the sequence/structure of the protein.

**Fig. 3:**
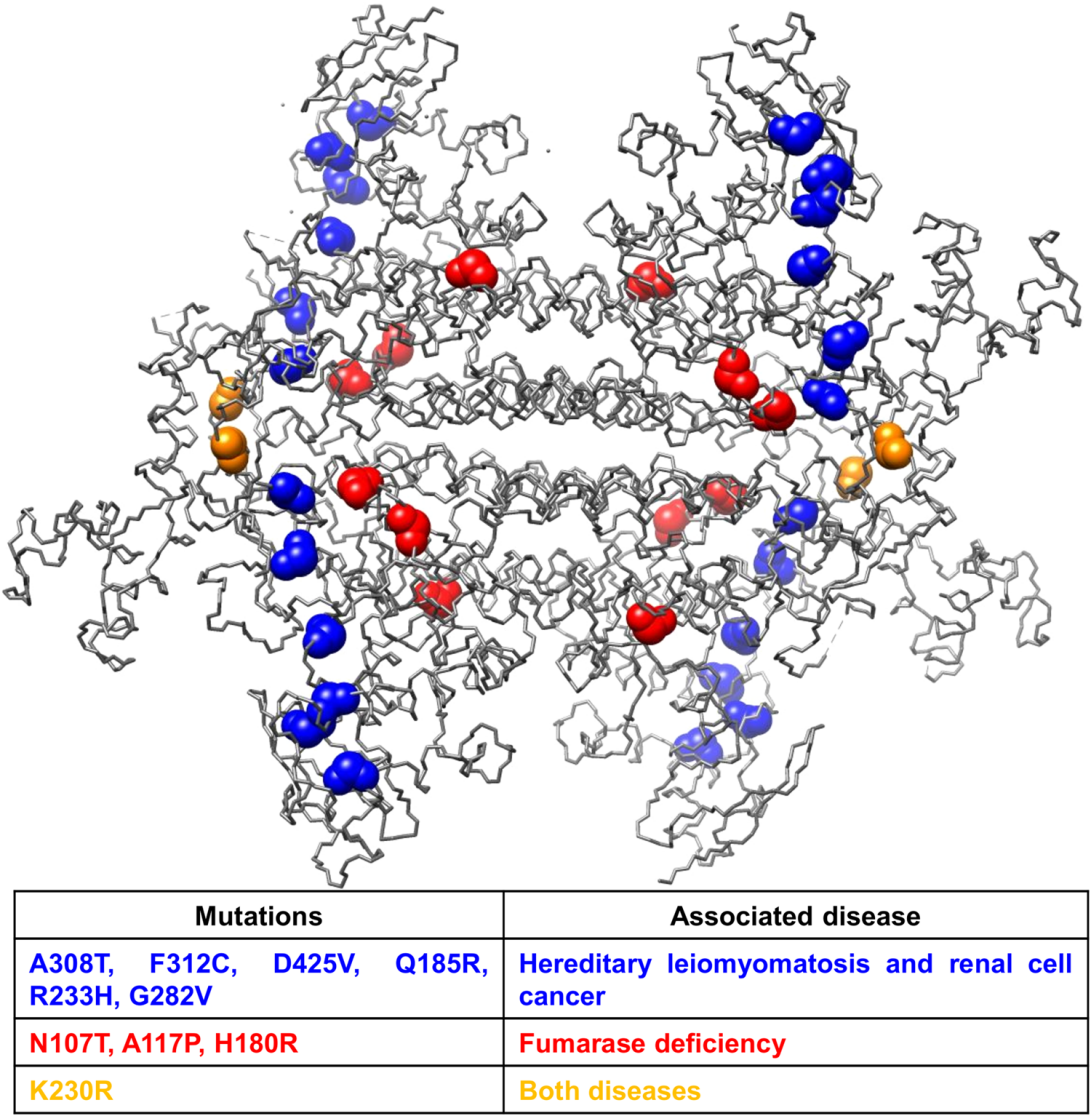
Structure of human fumarate hydratase tetramer (P07954). This protein has two associated diseases: fumarase deficiency (FD) and hereditary leiomyomatosis plus renal cell cancer (HLRCC). Marked in red are the mutations related to FD, and in blue are those related to HLRCC. Mutations associated with both diseases are in yellow

In our previous work on the bioinformatically prediction of multitasking proteins, we have suggested using some modelling programs to assign specific regions in the structure if this information is available [27, 28]. The method that was proposed to map the canonical and moonlighting functions in the structure of the protein (see Materials and Methods section), has been applied to the moonlighting protein Fumarase hydratase as an example (protein present in Table 1). This protein is associated with the fumarase deficiency (FD) and hereditary leiomyomatosis plus renal cell cancer (HLRCC) diseases. The UniProt database reports protein mutations involved in these diseases. Figure 3 shows the 3D structure of the tetramer as well as the mutations related to FD and HLRCC, which are depicted in red and blue, respectively. In yellow, the mutations associated with both diseases are highlighted. This picture clearly shows that the *canonical function, which is related to FD, is in the center of the tetramer, while the moonlighting* function, which is related to HLRCC, is in a different protein region. The structure and amino acid mutants strongly suggests the molecular basis of the disease, because these mutations seem to perturb the interaction and formation of the correct tetramer, which in turn can change indirectly, in certain degree, the precise positioning of the amino acids of the active center, reducing its activity.

As shown in the above examples, the mapping of each function on the 3D structure of a multitasking protein might be useful for the understanding of its normal and pathological functions.

## Conclusions

In summary, the fact that 78% of moonlighting proteins are involved in human diseases and 48% of them are targets of current drugs, suggests that moonlighting is not a rare phenomenon in proteins causing human diseases, and that their detailed study might explain some collateral drug effects.

## Electronic supplementary material

Below is the link to the electronic supplementary material.

Supplementary material 1 (XLS 321 KB)
